# Molecular and Cellular Mechanisms Involved in Host-Specific Resistance to Cyst Nematodes in Crops

**DOI:** 10.3389/fpls.2021.641582

**Published:** 2021-03-09

**Authors:** Qi Zheng, Vera Putker, Aska Goverse

**Affiliations:** Laboratory of Nematology, Department of Plant Sciences, Wageningen University, Wageningen, Netherlands

**Keywords:** cyst nematode, host-specific resistance, resistance gene, resistance locus, effector, plant immunity, immune receptor

## Abstract

Cyst nematodes are able to infect a wide range of crop species and are regarded as a major threat in crop production. In response to invasion of cyst nematodes, plants activate their innate immune system to defend themselves by conferring basal and host-specific defense responses depending on the plant genotype. Basal defense is dependent on the detection of pathogen-associated molecular patterns (PAMPs) by pattern recognition receptors (PRRs), while host-specific defense mainly relies on the activation of canonical and non-canonical resistance (*R*) genes or quantitative trait loci (QTL). Currently, application of *R* genes and QTLs in crop species is a major approach to control cyst nematode in crop cultivation. However, emerging virulent cyst nematode field populations are threatening crop production due to host genetic selection by the application of a limited set of resistance genes in current crop cultivars. To counteract this problem, increased knowledge about the mechanisms involved in host-specific resistance mediated by *R* genes and QTLs to cyst nematodes is indispensable to improve their efficient and sustainable use in field crops. Despite the identification of an increasing number of resistance traits to cyst nematodes in various crops, the underlying genes and defense mechanisms are often unknown. In the last decade, indebt studies on the functioning of a number of cyst nematode *R* genes and QTLs have revealed novel insights in how plants respond to cyst nematode infection by the activation of host-specific defense responses. This review presents current knowledge of molecular and cellular mechanisms involved in the recognition of cyst nematodes, the activation of defense signaling and resistance response types mediated by *R* genes or QTLs. Finally, future directions for research are proposed to develop management strategies to better control cyst nematodes in crop cultivation.

## Introduction

Cyst nematodes are notorious plant parasites infecting a broad range of crops worldwide. The most damaging species include soybean cyst nematode (SCN; *Heterodera glycines*), with more than US1.5 billion of economic losses each year in the United States alone; potato cyst nematode (PCN; *Globodera pallida* and *Globodera rostochiensis*), with an estimated yield loss of 9% worldwide; and cereal cyst nematode (CCN; *Heterodera avenae*), with yield losses up to 90% under nematode favorable environmental conditions (Jones et al., 2013). For long, the management of cyst nematode infections relied on the use of nematicides. Currently, cyst nematode control is highly dependent on crop rotation strategies and the application of a limited set of resistance genes in crop cultivars due to the ban on pesticide use in the soil since the early 00’s (Hillocks, 2012). However, the application of a limited repertoire of resistance genes has resulted in host genetic selection of resistance-breaking populations in the field (Turner and Fleming, 2002; Niblack et al., 2008; McCarville et al., 2017; Mwangi et al., 2019), which threatens the lifespan of current resistant crop cultivars. These concerns demand for new resistance resources as well as increased knowledge of the genes involved for the durable application of resistant crop cultivars in the future.

Cysts can persist in the soil for decades, which make them particularly difficult to control (Lilley et al., 2005; Jones et al., 2013). One female can produce hundreds of nematode eggs. When she dies her swollen body hardens into a cyst to protect eggs, allowing them to stay viable in the soil for many years in the absence of a proper host. Upon hatching from the eggs, the pre-parasitic second-stage juveniles (pre-J2) migrate through the soil in search of a suitable host plant. Upon entering the host plant roots, they move intracellularly through the root to establish a permanent feeding site near the vascular cylinder. In a susceptible host, a large, multinucleate feeding structure is formed through cell wall dissolution and fusion of neighboring cells, a so called syncytium. Cyst nematodes are fully dependent on this feeding structure for their development and reproduction as the syncytium is the only source of nutrients for this group of sedentary endoparasitic nematodes. Parasitic J2 develop through three molting steps into adult females when nutrients are abundantly available, but into adult vermiform males when this is not the case (Grundler et al., 1991). In resistant crop plants, however, cyst nematodes are unable to establish such a successful feeding relationship. Upon recognition of the infective cyst nematode juvenile, the development of a syncytium and subsequently the formation of cysts are prevented due to a local defense response. The application of resistant crop cultivars is therefore very effective, but only a limited set of resistance traits to control cyst nematodes is currently known. In addition, the genes responsible for cyst nematode resistance are identified and characterized for only a few single dominant resistance (*R*) genes or quantitative trait loci (QTL) like *Gpa2* (van der Vossen et al., 2000) in potato and *Rhg1* in soybean (Concibido et al., 2004). However, indebt studies on the functioning of the corresponding genes revealed novel insights in host-specific defense responses to cyst nematodes. Hence, they can serve as an example for other *R* genes and QTLs conferring resistance to cyst nematodes, for which this information is still lacking.

In this review, we explore the current knowledge on the molecular and cellular mechanisms involved in host-specific resistance against cyst nematodes as conferred by either single dominant *R* genes or QTLs. We first shortly address the plant immune system, including basal immunity against cyst nematodes. Then, we focus on host-specific resistance by addressing *R* gene-mediated defense responses, including effector-triggered immunity. We highlight how these *R* genes are able to recognize cyst nematodes and activate downstream defense responses in plant cells based on a few available model systems. Then, we briefly address how cyst nematodes are able to evade or suppress this type of host defense responses. We also specify the potential mechanisms of host-specific resistance mediated by QTLs, including non-canonical resistance phenotypes. Finally, we discuss how this knowledge may contribute to a better understanding of plant defense to cyst nematodes as well as the control of cyst nematodes in crop cultivation.

## Basal Defense Responses to Cyst Nematodes

During early stages in parasitism, cyst nematodes move intracellularly and cause root damage, leading to the release of Nematode-Associated Molecular Patterns (NAMPs) or Damage-Associated Molecular Patterns (DAMPs). These compounds can be perceived by extracellular Pattern Recognition Receptors (PRRs) and thereby elicit basal immunity, also named Pathogen-Associated Molecular Patterns (PAMPs)-Triggered Immunity (PTI; Choi and Klessig, 2016). To date, information about this first layer of plant immunity to cyst nematodes, including the role of NAMPs and PRRs, is limited. A conserved ascaroside (Asc#18) from *H. glycines* was identified as a NAMP as well as compounds present in cyst nematode (*Heterodera schachtii*) incubation water (NemaWater; Manosalva et al., 2015; Mendy et al., 2017). The *Arabidopsis* leucine-rich repeat (LRR) receptor-like kinase (RLK) NILR1 is yet the only classified cyst nematode PRR (Mendy et al., 2017). The activation of PTI results in a series of immune responses like reactive oxygen species (ROS)/NO production, secondary metabolite production, reinforcement of cell walls, and cell death around the migratory tract. For more details on cyst nematode-elicited PTI responses as well as the underlying molecular mechanism, we refer the reader to Sato et al. (2019).

Although PTI responses slow down nematode invasion and contribute to an effective defense in non-host plants, they are insufficient to stop the nematode from successfully infecting susceptible host plant roots. Just like other pathogens, nematodes are able to overcome PTI by the secretion of effector proteins which suppress basal immune responses (also called effector-triggered suppression or ETS; Jones and Dangl, 2006). Such cyst nematode effectors include GrVAP1, RHA1B, Ha18764, and GrCEP12 (Lozano-Torres et al., 2012; Chen et al., 2013; Kud et al., 2019; Yang et al., 2019). Most effectors are synthesized in esophageal gland cells and are secreted into the plant *via* a needle-like structure named the stylet (Hewezi and Baum, 2013; Vieira and Gleason, 2019). The suppression of basal immune responses enables cyst nematodes to establish a successful feeding relationship with a susceptible host plant for their development and reproduction (Ali et al., 2017; Vieira and Gleason, 2019).

## Effector-Triggered Defense Responses to Cyst Nematodes

In response to ETS, plants have evolved a second layer of immunity according to the zigzag model (Jones and Dangl, 2006). Resistant plant genotypes exhibit single dominant *R* genes, encoding immune receptors that recognize specific pathogen effectors or their activities, and subsequently activate so called effector-triggered immunity (ETI). Recognition can either be direct or indirect, meaning that a host-derived co-factor is required for successful pathogen perception (Jones and Dangl, 2006; Araújo et al., 2019). Moreover, *R* genes can only recognize their matching effector which is encoded by a corresponding avirulence (*Avr*) gene. Only when an *Avr* gene containing pathotype matches an *R* gene containing plant genotype, the plant can successfully activate a host-specific resistance response. This is known as the gene-for-gene concept (Flor, 1971), which also applies to cyst nematodes as demonstrated for the single dominant *R* gene *H1* from potato that confers host-specific resistance to avirulent populations of *G. rostochiensis* (Janssen et al., 1991).

### *R* Genes Against Cyst Nematodes Encode Different Types of Plant Immune Receptors

**Figure 1:**
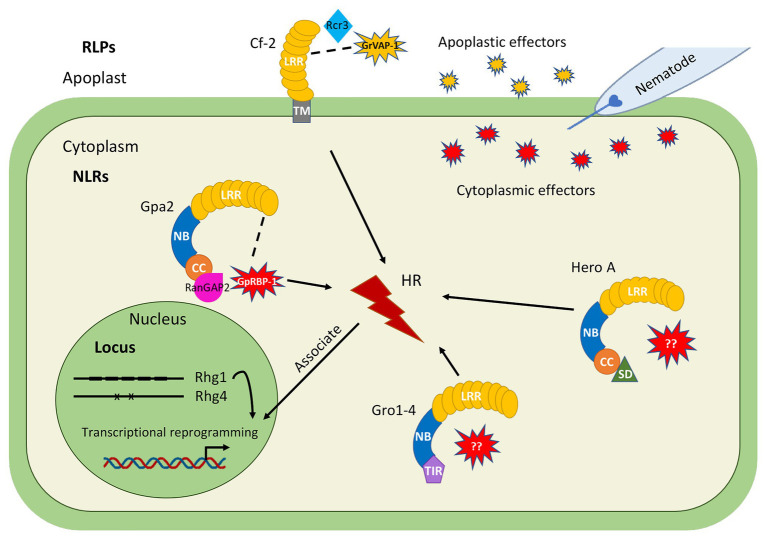
Overview of major cyst nematode resistance (*R*) genes and loci (QTLs) identified in crop species, for which knowledge is available on the molecular and cellular mechanisms underlying cyst nematode resistance. Extracellular immune receptors: *Cf-2* from tomato encodes a RLP and confers resistance to the potato cyst nematode (PCN) *Globodera rostochiensis*, respectively. Cf-2 detects the nematode effector GrVAP-1 *via* the host factor Rcr3, which is activated by apoplastic serine proteases named subtilases to induce apoplastic immunity. Intracellular Nucleotide binding (NB)-Leucine-rich Repeat (LRR; NLR) immune receptors: *Gro1-4* from potato encodes a toll-interleukin receptor-nucleotide binding-LRR (TIR-NB-LRR) protein, whereas *Gpa2* from potato encodes a coiled coil-NB-LRR (CC-NB-LRR) protein. *Hero A* from tomato encodes a Solanaceae Domain-CC-NB-LRR (SD-CC-NB-LRR) protein. These intracellular immune receptors all confer resistance to specific PCN populations from *G. rostochiensis*, *Globodera pallida*, or both. Only for Gpa2, the matching nematode effector GpRBP-1 is known which is able to activate a local HR response. Detection of nematode effector GpRBP-1 by Gpa2 requires a host factor RanGAP2. Resistance loci: the *Rhg1* and *Rhg4* loci from soybean confer resistance to field populations from the cyst nematode *Heterodera glycines*. *Rhg1*-mediated resistance depends on copy number variation, as the high copy number *Rhg1* type confers resistance on its own while the low copy number *Rhg1* type requires the *Rhg4* locus to confer resistance. Two polymoriphisms determine *Rhg4*-mediated resistance.

Effector detection by immune receptors occurs intra- and extracellularly. Extracellular immune receptors, including RLKs and receptor-like proteins (RLPs), contain a LRR domain fused to a transmembrane domain (Takken and Joosten, 2000; Kanyuka and Rudd, 2019). Extracellular immune receptors encoded by *R* genes have the same type of structure as PRRs (Boutrot and Zipfel, 2017), but they are able to activate highly specific defense responses upon the direct or indirect detection of apoplastic effectors from specific pathogen strains. In terms of resistance to cyst nematodes, so far two RLP immune receptors have been characterized (Figure 1). One example is the sugar beet receptor Hs1^pro-1^, which was linked to resistance to the cyst nematode *H. schachtii* (Cai et al., 1997). However, the resistance phenotype cannot be inherited to the next generations by backcrossing of an *Hs1^pro-1^* genotype and a susceptible genotype (Sandal et al., 1997) raising questions about its contributions to cyst nematode immunity in sugar beet. Another example is the tomato immune receptor Cf-2, which confers resistance to the PCN *G. rostochiensis* (Lozano-Torres et al., 2012).

The most abundant class of *R* genes encodes intracellular Nucleotide binding (NB)-LRR proteins (NLR). NLRs contain a C-terminal LRR (LRR) domain involved in recognition and a central nucleotide-binding, Apaf-1, R-proteins, and CED-4 (NB-ARC) domain, which acts as a molecular switch and consists of three subdomains (NB, ARC1, and ARC2). The N-terminus is a signaling domain, which divides NLRs into either a subclass of Toll-interleukin receptor (TIR)-like receptors (TIR-NB-LRRs) or coiled coil (CC) receptors (CC-NB-LRRs). Both subclasses are found to be encoded by *R* genes conferring resistance against cyst nematodes (Figure 1). For example, the potato resistance gene *Gpa2* confers resistance to *G. pallida* and its product belongs to the CC-NB-LRR (CNL) type (van der Vossen et al., 2000), whereas a typical example of a TIR-NB-LRR (TNL) is encoded by *Gro1-4* from potato conferring resistance to *G. rostochiensis* (Paal et al., 2004). In addition, some CNLs exhibit an extended Solanaceae Domain (SD) at the N terminus of the CC domain, which is uniquely found in Solanaceous plant species. For example, the tomato resistance gene *Hero A* is a typical example that belongs to the SD-CNL type, which confers broadspectrum resistance to PCN populations from *G. pallida* and *G. rostochiensis* (Ernst et al., 2002).

### Molecular Mechanisms of Different Types of Cyst Nematode *R* Genes

A major bottleneck in our understanding of *R* gene-mediated cyst nematode resistance is that the matching effector for most nematode *R* genes is unknown. Currently, only two *R* gene-effector pairs are identified. These are the extracellular immune receptor Cf-2, which elicits an immune response upon recognition of the effector GrVAP1 from *G. rostochiensis* (Lozano-Torres et al., 2012) and the intracellular immune receptor Gpa2, which recognizes the effector GpRBP-1 from *G. pallida* (Sacco et al., 2009). Over the last decade, several indebt studies have revealed novel insights in their functioning and role in conferring host-specific resistance to cyst nematodes. Therefore, *Cf-2* and *Gpa2* can serve as examples for other cyst nematode resistance genes, for which information on the molecular mechanisms underlying recognition and downstream signaling activation of immune responses against cyst nematodes is still lacking.

#### Cf2-Mediated Apoplastic Immunity to Cyst Nematodes

The extracellular immune receptor Cf-2 belongs to the RLP type of immune receptors and confers apoplastic immunity to the PCN *G. rostochiensis* upon detection of the effector GrVAP1, which is produced in the subventral glands during the onset of parasitism (Lozano-Torres et al., 2012). The detection of GrVAP1 by Cf-2 is indirect through the detection of perturbations of the apoplastic papain-like cysteine protease (PLCP) Rcr3. Cf-2 detection of GrVAP1 through Rcr3 results in the activation of cyst nematode resistance, which induces a local programmed cell death response in cells directly around the nematodes as well as in most of the nematode-induced feeding structures (Lozano-Torres et al., 2012). A recent study shows the underlying mechanism of how Rcr3 in tomato participates in the activation of defense responses. Rcr3 is present in its inactive form ProRcr3, and a group of proteases called subtilases cleave off the prodomain of Rcr3. This results in a mature mRcr3, thereby creating a binding site for the effector (Paulus et al., 2020). Since distantly related subtilases can also activate Rcr3 in *Nicotiana benthamiana*, this suggests that there might be a network of proteolytic cascades in Solanaceous plants to provide robust apoplastic immunity (Kourelis et al., 2020; Paulus et al., 2020), which may also apply to cyst nematodes in Cf-2 resistant tomato plants.

Interestingly, GrVAP1 is not the only pathogen effector that targets Rcr3. Avr2, from the fungus *Cladosporium fulvum*, Cip1 from the bacteria *Pseudomonas syringae*, and several EPIC effectors from the oomycete *Phytophthora infestans* target Rcr3 as well with a variable prosperity in Cf-2-mediated defense responses (Rooney et al., 2005; Ilyas et al., 2015; Misas Villamil et al., 2019). However, these effectors also inhibit a paralog of Rcr3: Pip1 which is present more abundantly compared to Rcr3. Findings by knockdown studies (Ilyas et al., 2015) suggested that Pip1 is the actual operative target of the effectors, while Rcr3 acts as a decoy to trap the pathogen into a recognition event. This guard/decoy-recognition model allows plants to respond faster and more efficient to multiple and unrelated pathogens present in the environment *via* a common host target (van der Hoorn and Kamoun, 2008). Moreover, it indicates that different pathogens including cyst nematodes have evolved effectors that are able to inhibit plant proteases during co-evolution (Kourelis et al., 2020).

#### Gpa2-Mediated Intracellular Immunity to Cyst Nematodes

In contrast to Cf-2, the intracellular CNL immune receptor Gpa2 activates a specific defense response upon detection of the dorsal gland effector GpRBP-1 from *G. pallida* inside the cell, resulting in a hypersensitive response (HR) in *N. benthamiana* leaves in agroinfiltration assays (Sacco et al., 2009). GpRBP-1 recognition by Gpa2 is determined by a single amino acid polymorphism at position 187 in the SPRY domain of GpRBP-1 (Sacco et al., 2009). However, no physical interaction between Gpa2 and GpRBP-1 was detected which may point at an indirect interaction. It is hypothesized that Gpa2 senses the presence of GpRBP-1 *via* a host factor RanGAP2, due to a physical interaction between the CC domain of Gpa2 and RanGAP2 (Sacco et al., 2007, 2009). Silencing of RanGAP2 compromises Gpa2-mediated HR, but artificial tethering of RanGAP2 and GpRBP-1 enhances Gpa2-mediated defense responses (Sacco et al., 2009). These data suggest that RanGAP2 potentially works as a recognition co-factor for Gpa2 and may play an important role in downstream signaling regulation.

Remarkably, RanGAP2 is also required for Rx1-mediated resistance responses. The CNL Rx1 is a close homolog of Gpa2 that resides in the same *R* gene cluster on ChrXII of potato and confers resistance to *Potato virus X* (van der Vossen et al., 2000). The CC domain of Rx1 interacts with the N-terminal WPP domain of RanGAP2 and is present in plant cells as a heteromeric complex when in its inactive state (Sacco et al., 2007; Hao et al., 2013). Moreover, Rx1 locates in both the nucleus and the cytoplasm of plant cells, and RanGAP2 acts as a cytoplasmic retention factor of Rx1, thereby facilitating Rx1 functioning (Tameling et al., 2010). The nuclear hyperaccumulation of Rx1 mediated by nuclear targeted RanGAP2 WPP domain blocks Rx1 auto-activity. As the Gpa2, CC domain also interacts with RanGAP2, it is speculated that hyperaccumulation of Gpa2 in the nucleus may also block its defense signaling initiation. Nonetheless, whether RanGAP2 also regulates Gpa2 functioning by mediating its subcellular partitioning remains to be demonstrated.

Structure-informed studies revealed the contribution of intra- and interdomain interactions in Gpa2 functioning as a molecular switch in plant immunity to cyst nematodes. Extensive sequence exchange between Gpa2 and Rx1 showed that a minimal region of the ARC2 together with the N-terminal repeats of the LRR domain is sufficient to initiate activation of the immune receptors (Slootweg et al., 2013). Additionally, domain swaps between regions of the LRR of Gpa2 and Rx1 resulted in the conversion of virus resistance into nematode resistance and vice versa (Slootweg et al., 2017), demonstrating that the CC-NB-ARC domain operates independently of the pathogen that is recognized whereas the LRR domain determines recognition specificity. Furthermore, comparative sequence analysis and computational structure analysis revealed that Rx1/Gpa2 polymorphisms in the LRR domain are under positive selection and surface exposed consistent with a possible role in pathogen detection (Slootweg et al., 2013). However, the dynamic process of how *R* genes like Gpa2 switch from an inactive to an active state upon cyst nematode detection remains elusive. Breakthrough discoveries on the 3D modeling and cryoEM-structure analyses as reported for the *Arabidopsis* CNL immune receptor ZAR1 in its inactive, primed, and activated state (Wang et al., 2019a,b) are expected to provide novel insights in the functional dynamics of NLR immune receptors in the near future. Moreover, it underscores the importance of structural approaches in plant resistance research to increase our understanding about the molecular warfare between cyst nematodes and their host plants.

Recently, both Rx1 and Gpa2 have been identified as so called sensor NLRs (Wu et al., 2017; Adachi et al., 2019a). In Solanaceous plants, a major class of CNLs, have been identified to form an immunoreceptor network in which sensor NLRs can directly or indirectly perceive molecules derived from pathogens, but require paired so called helper NLRs to activate immune responses. As a helper, NRCs (NLR required for cell death) are thought to translate upstream signaling from sensor NLRs to downstream signaling components for the activation of immune responses (Wu et al., 2018; Adachi et al., 2019b). Rx1 requires NRC2, NRC3, or NRC4 to activate a resistance response. The triple silencing of NRC2, NRC3, and NRC4 compromises Rx1-mediated resistance to PVX while the individual silencing remains Rx1 functional, indicating that these NRC proteins redundantly contribute to Rx1-mediated resistance. It remains to be seen which helper NRCs are required for the activation of downstream defense response to cyst nematodes mediated by sensor NLRs like Gpa2 as well as Hero A (Wu et al., 2017). NRCs including NRC4 carry a MADA motif at the N-terminus, which is sufficient for triggering cell death (Adachi et al., 2019a). Interestingly, the MADA motif does not exist in NRC-dependent sensor NLRs like Rx1 and Gpa2, suggesting this motif might be degenerated during evolution. It is therefore likely that Rx1 and Gpa2 rely on the MADA motif of their helper NRCs to activate defense responses upon virus and nematode recognition, respectively.

### Downstream Signaling Pathways Involved in Host-Specific Resistance to Cyst Nematodes

Activation of R proteins leads to the transcriptional reprogramming of cells leading to the activation of local and systemic defense responses. Transcriptome studies revealed insights in downstream signaling pathways involved in host-specific resistance to cyst nematodes (Uehara et al., 2010; Walter et al., 2018). For instance, in resistant tomato harboring the *Hero A* gene, the salicylic acid (SA)-dependent pathogenesis-related protein 1 (PR-1) shows a markable increase in expression at 3 dpi upon *G. rostochiensis* infection compared to susceptible plants (Uehara et al., 2010). This was not the case in resistant plants with an extra inserted *NahG* gene, which can prevent SA accumulation, indicating that SA plays a key role in Hero A-mediated resistance to cyst nematodes. Another transcriptome study performed on resistant potato containing the *H1* gene shows upregulation of many genes after *G. rostochiensis* infection, including the tomato stress-responsive factor TSRF1 and a cysteine protease (Walter et al., 2018). TSRF1 is an ethylene responsive factor, which can be upregulated by ethylene or SA treatment (Zhang et al., 2004). The same study also shows that TSRF1 can interact with the GCC box located in the promoter of *PR* genes. Taken together, H1-induced TSRF1 upregulation possibly triggers PR proteins accumulation, which is consistent with the finding of systemic PR protein accumulation in leaves in *G. rostochiensis* infected H1 resistant potato plants (Hammond-Kosack et al., 1989). From this, a picture emerges in which ethylene- and/or SA-dependent pathways might be involved in H1-triggered resistance to cyst nematodes similar to what has been reported for other *R* gene-mediated resistance responses to other pathogens (Denancé et al., 2013; Broekgaarden et al., 2015; Islam et al., 2019).

Transcriptome analysis on the resistant wheat genotype VP1620 shows that jasmonic acid (JA) regulated PR4 and PR10 are significantly induced upon infection by CCN *H. avenae*, suggesting that the JA pathway is involved in resistance against CCN in this monocot crop (Kong et al., 2015). Moreover, the abundant presence of phospholipase D1/2 in the KEGG pathways suggests a role for ROS in conferring resistance to CCN (Kong et al., 2015), since phospholipase positively regulates defense responses *via* the ROS pathway (Wang, 2005; Pinosa et al., 2013). In another transcriptome study, transcription factor WRKY40 and WRKY70 are upregulated in resistant soybean genotype PI533561 upon infection by a *H. glycines* virulent type named HG type 0 (Jain et al., 2016). WRKY70 has been shown to be involved in the regulation of the ROS pathway in defense, suggesting that the ROS pathway might be involved in resistance to SCN as well. Moreover, the PR-5 like receptor kinase shows an upregulation in the resistant genotype, indicating that a similar SA pathway may be involved in response to SCN infection. Interestingly, WRKY40/70 and one PR family protein (Phvul.005G081500) also show an upregulation in susceptible genotype GTS-900 (Jain et al., 2016); implying ROS and SA pathways may also be involved in basal defense.

### *R* Gene-Mediated Resistance Response Types to Cyst Nematodes

**Figure 2:**
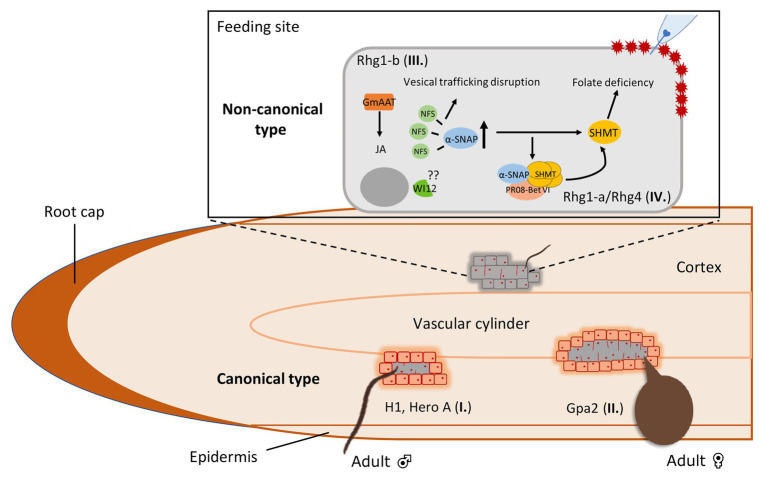
Host-specific defense responses against cyst nematodes in plant roots harboring major *R* genes or resistance loci (QTLs). Type I. A subset of *R* genes mediate early defense responses to cyst nematodes that allows syncytium initiation but restricts further expansion by forming a layer of necrotic cells around the young feeding structures. As a result, these encapsulated syncytia allow the development of males but not females. This “male-biased” resistance is seen for *R* genes like *H1* in potato and *Hero A* in tomato. Type II. Another subset of *R* genes mediates a late defense response that allows syncytium formation and expansion, supporting the development of females. In this case, a layer of necrotic cells is formed around the expanded syncytium which disrupts the connection between the syncytium and the vascular cylinder. Thereby, female development is restricted due to the lack of nutrition. This “female-biased” resistance is observed for *R* genes like *Gpa2* from potato. Whereas the type I and II responses are typical for cyst nematode *R* genes encoding NB-LRR immune receptors, the resistance loci *Rhg1* and *Rhg4* trigger non-canonical resistance responses to cyst nematodes in soybean roots. Type III. The high copy number *Rhg1-b* type encodes a *α*-SNAP protein that poorly interacts with the protein NSF to disrupt vesical trafficking. Meanwhile, the hyperaccumulation of α-SNAP is thought to promote the collapse of the plant-nematode biotrophic interface leading to nematode resistance. Type IV. *Rhg4*-mediated resistance is determined by two polymorphisms in the encoded protein serine hydroxymethyltransferase (SHMT). SHMT regulates folate homeostasis leading to folate deficiency, causing the poor development and difficult maintenance of syncytia. Interestingly, cross-talk occurs in low copy number *Rhg1-a*, which requires *Rhg-4* to activate SCN resistance. It is thought that the elevated levels of *Rhg1-a* encoded α-SNAP induces *Rhg-4* encoded SHMT, which then physically interact with each other to form a complex with the pathogenesis-related protein PR08-Bet. This multiprotein complex regulates the activity of SHMT which leads to syncytium collapse and thus cyst nematode resistance.

In resistant plants, cyst nematodes are blocked in their life cycle and reproduction due to host-specific defense responses induced by R proteins. During normal cyst nematode development and reproduction, the syncytium is crucial since it functions as the only nutrient source for this obligatory biotrophic endoparasite. Host-specific resistance by *R* genes often associates with an HR, which causes necrosis around the nematode-induced syncytia. According to the timing in the ontogeny of feeding structures and characteristic cytological features, host-specific resistance to cyst nematodes can be roughly divided into two types (Goverse and Smant, 2014; Smant et al., 2018). The first type allows the initiation of a syncytium, but the expansion of the syncytium is restricted by the formation of a layer of necrotic cells around the young feeding structure (Figure 2). The initiated syncytium still allows the development of males, but does not support the development of females due to the poorly developed feeding structure. This type of “male-biased” resistance is observed for *R* genes like *H1* and *Hero A* (Rice et al., 1985; Sobczak et al., 2005). The second type of resistance occurs in a later stage of the plant-nematode interaction and allows syncytium formation and expansion (Figure 2). These young syncytia are functional and support the initiation of female development. However, a layer of necrosis around the syncytium is formed soon thereafter to disconnect the syncytium from the vascular cylinder. In this way, the transfer cell function of the syncytium is compromised and female development is arrested due to starvation. A typical example of this delayed resistance responses are induced by the potato R protein Gpa2, which confers host-specific resistance to PCN *G. pallida* (van der Vossen et al., 2000; Mwangi et al., 2019).

## Evasion or Suppression of *R* Gene-Mediated Immunity by Cyst Nematode Effectors

To evade recognition by R proteins, cyst nematodes have evolved effector variants which are not recognized or able to suppress the activation of host-specific defense responses. Examples of such effectors were recently identified, such as SPRYSEC effectors (Diaz-Granados et al., 2016), an E3 ubiquitin ligase RHA1B (Kud et al., 2019), and an expansin-like protein GrEXPB2 (Ali et al., 2015a). The SPRYSEC effector family is characterized as a single SPRY domain-containing protein, secreted from the dorsal esophageal gland of PCNs (Diaz-Granados et al., 2016). SPRYSEC effectors from *G. rostochiensis*, including SPRYSEC-4/5/8/18/19, function as suppressors of HR and disease resistance mediated by CNL immune receptors like Rx1 (Postma et al., 2012; Ali et al., 2015b). A typical example is *G. rostochiensis* effector SPRYSEC-19, which can physically interact with the tomato intracellular CNL immune receptor homolog Sw5F both *in vitro* and *in planta* as show by Y2H; GST-pull down and Co-IP (Rehman et al., 2009; Postma et al., 2012). The minimal domain for this interaction is the C-terminal end of the LRR domain. Although this is in line with a possible role for the LRR in cyst nematode detection, co-expression of Sw5F and SPRYSEC-19 did not trigger an HR or resistance response to nematodes. Therefore, the function of Sw5F in plant immunity to cyst nematode still remains elusive (Postma et al., 2012).

Another example is the effector from *G. pallida* named RHA1B, which is characterized as an E3 ubiquitin ligase that functions in ubiquitin-proteasome pathway-mediated protein degradation (Kud et al., 2019). RHA1B applies two distinct ways to suppress plant immunity during nematode parasitism: it suppresses PTI signaling *via* a yet unknown E3-independent manner, and it suppresses HR mediated by several R proteins *via* E3-dependent degradation of R proteins. In addition, an apoplastic effector from *G. rostochiensis*, an expansin-like protein GrEXPB2, could inhibit a set of NLR immune receptors, including Rx1 and N mediated defense responses in the cytoplasm (Ali et al., 2015a). As GrEXPB2 is highly accumulating in pre-parasitic stages and decreases quickly during plant infection, it is speculated that GrEXPB2 might be involved in suppression of early PTI or ETI upon root invasion by cyst nematodes. However, GrEXPB2 also triggers necrosis in tomato and potato but not tobacco (Ali et al., 2015a). The activation of defense in a plant species-dependent manner suggests the specific recognition of the effector by the plant immune system, indicating that GrEXPB2 retains its dual role in suppressing and triggering plant defense responses.

## Quantitative Trait Loci Conferring Host-Specific Resistance to Cyst Nematodes

In addition to single dominant *R* genes that encode for immune receptors, host-specific resistance to cyst nematodes can also be conferred by QTLs. In the last decades, many QTLs to different cyst nematode species in various major food crops have been identified such as *Rhg1* and Rhg4 to SCN in soybean (Cook et al., 2012; Liu et al., 2012), *Rha2* from barley conferring resistance to CCN (Kretschmer et al., 1997), and *Gpa*, *Grp1*, and *Gpa5* among others in potato conferring resistance to PCN (Kreike et al., 1994; van der Voort et al., 1998, 2000). QTLs may involve multiple or polymorphic genes present at different loci or even a particular locus, including essential genes that are required for achieving nematode resistance. Often, QTLs co-localize with NLR gene clusters as observed in potato suggesting that classical resistance gene homologs may contribute to host-specific resistance mediated by these QTLs (Bakker et al., 2011). However, an alternative explanation for the quantitative behavior of these QTLs loci could be the result of the heterogeneous composition of cyst nematode field populations used in the resistance tests. Interestingly, in case of *Rhg1* and *Rhg4*, host-specific resistance is not linked to NLR-mediated immunity and was shown to be mediated by non-canonical resistance genes (Cook et al., 2012; Liu et al., 2012).

Recently, several studies have revealed first insights in the molecular mechanisms underlying this novel type of resistance to cyst nematodes (Figure 1). For *Rhg1*, copy number variation plays an important role in determining SCN resistance since a higher copy number of genes at the *Rhg1* locus relates to an increased resistance phenotype (Cook et al., 2012, 2014; Yu et al., 2016). A copy of a 31-kilobase segment carrying three genes that contribute to resistance leads to an increased resistance to SCN, but only when the expression of this set of genes increases at the same time. Based on the copy number, at least two classes of *Rhg1* haplotypes are identified: low-copy *rhg1* (*rhg1-a*, Peking-type) and high-copy *rhg1* (*rhg1-b*, PI 88788-type). Three genes located in the 31-kilobase segment are required for resistance, which encode for an amino acid transporter, an alpha-soluble NSF (N-ethylmaleimide–sensitive factor) attachment protein (α-SNAP) protein and a wound-inducible domain (WI12) protein (Cook et al., 2012; Guo et al., 2019). The *rhg1-a* and *rhg1-b* haplotypes encode two different α-SNAP variants (Liu et al., 2017; Bayless et al., 2019), but both variants hyperaccumulate at SCN infection sites (Bayless et al., 2016, 2019; Lakhssassi et al., 2020a). The *rhg1-a* encoded α-SNAP carries a copia retrotransposon in its structure named RAC (*Rhg1* α-SNAP copia), which harbors intrinsic transcriptional activity (Bayless et al., 2019). However, the exact role of RAC in SCN resistance is not clear yet as no direct effect of RAC in regulating Peking-type *GmSNAP18* mRNA and protein level is detected. Different from the Peking-type GmSNAP18, *rhg1-b* encoded α-SNAP lacks the RAC element in its structure (Bayless et al., 2019). Interestingly, PI 88788-type GmSNAP18 poorly interacts with the NSF protein and disrupts vesical trafficking (Bayless et al., 2016). As such, the hyperaccumulation of PI 88788-type GmSNAP18 is thought to promote the collapse of the plant-nematode biotrophic interface leading to nematode resistance (Figure 2). The role of the amino acid transporter *Rhg1*-GmAAT in SCN resistance may be *via* the JA pathway, as overexpression of *Rhg1*-GmAAT induces JA accumulation and glutamic acid tolerance (Guo et al., 2019). The role of WI12 in *rhg1* mediated SCN resistance is not understood yet.

*Rhg4* is another non-canonical resistance locus and *Rhg4*-mediated resistance to SCN is determined by two genetic polymorphisms residing near the ligand-binding sites of a serine hydroxymethyltransferase (SHMT; Liu et al., 2012). Mutation analysis showed that these polymorphisms affect the enzymatic activity of SHMT, which ubiquitously exists in nature and exhibits a key role in one-carbon folate metabolism (Cossins and Chen, 1997). The change of folate homeostasis can lead to folate deficiency, which may cause the poor development and difficult maintenance of syncytia. Alternatively, folate deficiency may trigger HR-like programmed cell death of the syncytium and lead to the death of nematodes as nematodes get insufficient folate from the host plant. The findings of *Rhg4*-mediated resistance reveal that plants may disrupt developmental or metabolic processes of the feeding structure itself to achieve resistance (Figure 2). The functional study of mutations on the SHMT protein structure revealed key residues that affect resistance to SCN (Kandoth et al., 2017; Shaibu et al., 2020). Meanwhile, the SHMT mutant still exhibits other functions in addition to its main enzymatic role in SCN resistance.

*Rhg4* is required for Peking-type *rhg1-a* to confer resistance to SCN successfully (Liu et al., 2012) and this involves both Peking-type *rhg1-a* GmSNAP18 and *Rhg4-a* GmSHMT08 (Liu et al., 2017). Interestingly, two recent studies have revealed novel insights in the mechanisms underlying the crosstalk between Peking-type *rhg1-a* GmSNAP18 and *Rhg4-a* GmSHMT08. Peking-type GmSNAP18 physically interacts with the *Rhg4-a* GmSHMT08 tetramer and this interaction is strengthened by the pathogenesis-related protein GmPR08-Bet VI (Lakhssassi et al., 2020a,b). Mutational analysis shows that GmSHMT08 tetrameric structure is essential for GmSNAP18/GmSHMT08/GmPR08-Bet VI multi-protein complex formation (Lakhssassi et al., 2020b). GmPR08-Bet VI is suggested to contribute in SCN resistance as overexpression of the gene leads to an enhanced resistance to SCN while mutation of cytokinin-binding sites of the gene product abolishes its effect on SCN resistance (Lakhssassi et al., 2020a). Furthermore, these studies indicate that SA, cytokinin, and ROS pathways are involved in SCN resistance. Under SA and cytokinin treatments, GmSNAP18 and GmPR08-Bet VI are induced while GmSHMT08 is only induced in the presence of GmSNAP18, implying GmSHMT08 functions downstream of GmSNAP18 (Lakhssassi et al., 2020a). Collectively, a picture emerges in which SCN infection induces increased expression of GmSNAP18 which leads to a subsequent induction of GmSHMT08. Next, a GmSNAP18/GmSHMT08/GmPR08-Bet VI multi-protein complex is formed to regulate activity of GmSHMT08, including one-carbon folate metabolism and redox metabolism maintenance. Also, the trafficking of GmPR08-Bet VI toward infected cells increases the cytotoxicity in the cells. Consequently, necrosis and disruption of the syncytium occurs (Figure 2). Interestingly, for PI 88788-type of resistance, GmSNAP18 also interacts with GmSHMT08 and GmPR08-Bet VI is able to strengthen this interaction in the cells as in Peking-type (Lakhssassi et al., 2020b). However, PI 88788-type GmSNAP18 shows the incompatibility with Peking-type GmSHMT08 and this may explain the current difficulty in applying soybean lines that combine PI 88788-type and Peking-type in breeding strategies.

## Conclusion and Future Perspectives

In this review, we have highlighted the current knowledge on the molecular and cellular mechanisms involved in host-specific resistance against cyst nematodes based on a few well-studied examples. In addition, information from other studies on the activation of host-specific downstream defense responses were addressed as well and integrated in this review to provide a comprehensive picture of the molecular mechanisms underlying different types of cyst nematode resistance known to date. From this, it can be concluded that host-specific defense to cyst nematodes in most cases is conferred by classical *R* genes encoding extra- and intracellular immune receptors upon specific recognition of cyst nematode effectors or their activities in the plant cell. Interestingly, host-specific defense to cyst nematodes is also conferred by non-canonical resistance loci directly interfering in the biotrophic interface between nematodes and their host plants. To counteract host-specific defense responses, either mediated by typical single dominant *R* genes as well as non-canonical QTLs, cyst nematodes have evolved molecular mechanisms to evade recognition by the plant immune system or by active suppression of defense responses. This co-evolutionary arms race explains both the diversity and copy number variation observed in *R* gene homologs as well as effector variants similar to what is reported for other pathosystems.

For cyst nematode *R* genes, only two model systems (e.g., Gpa2/GpRBP-1 and Cf2/GrVAP-1) are currently available for which the matching immune receptor-effector pair is known. Such pairs allow the performance of biochemical, cellular, molecular, and functional studies to resolve the mechanisms underlying plant immunity to cyst nematodes. For example, agroinfiltration assays on leaves of *N. benthamiana* can be used instead of time consuming and highly variable nematode infection assays on crop plants, for which often effective genetic tools are lacking. However, only a few cyst nematode *R* genes and resistance loci are identified and characterized to date, which hampers advances in the field. It is anticipated though that this number will increase in the near future as the result of sequencing efforts of genomic regions combined with refined genetic mapping approaches in various crops linked to cyst nematode resistance (Concibido et al., 2004; Banu et al., 2017) For example, cyst nematode *R* gene loci are known in crops for years, such as the *H1* and *H2* gene in potato (Ellenby, 1952; Blok and Phillips, 2012), but the genes responsible for the resistance are still unknown. However, with the tools currently available for genetical and functional genomics studies, it is possible to identify the causal genes. Fine mapping in combination with genome sequencing revealed that the *H1* locus harbors a cluster of NLR candidate genes, suggesting that the *H1* gene is a classical single dominant *R* gene (Finkers-Tomczak et al., 2011). Similarly, the recent mapping of the *H2* gene in combination with NLR-specific enrichment sequencing (RenSeq), Diagnostic resistance gene enrichment (dRenSeq), and Generic-mapping enrichment sequencing of single/low-copy number genes (GenSeq) also revealed candidate *R* genes encoding NLR immune receptors (Strachan et al., 2019). So, the next step is to show their contribution to cyst nematode resistance, which can be examined by for example RNA interference (RNAi) or CRISPR-Cas9 gene editing techniques in a resistant background followed by infection of plants with a matching avirulent cyst nematode population to see if *R* gene mediated resistance is compromised (Shaibu et al., 2020).

The application of these advanced sequencing techniques combined with molecular mapping approaches will not only enhance the identification of novel *R* genes to cyst nematodes in major crops like potato, but also provides the accurate positioning of these genes on the chromosomes of crop genomes. Together, this knowledge and knowhow will facilitate the improvement of crop resistance to cyst nematodes by marker assisted selection and molecular breeding. Functional characterization of the (novel) responsible genes or QTLs as well as detailed insights about the underlying molecular and cellular mechanisms can contribute to the rational design of novel *R* genes (loci) with different recognition spectra, either through gene editing or targeted selection of resistance gene homologs in natural or breeding populations. In this way, broad spectrum resistance could be achieved in crop species to different cyst nematode populations. Also the stacking of *R* genes with different recognition spectra provides a promising strategy to obtain broad spectrum and durable resistance to cyst nematodes.

Another major bottleneck in this research area is the lack of knowledge on the corresponding effectors recognized by known cyst nematode *R* genes like *Hero A* or *GroV1*. Over the last decades, an increasing number of cyst nematode effectors have been identified and characterized, including several suppressors of plant immunity. However, whether certain effector variants are recognized by the plant immune system remains to be demonstrated. Gland-specific sequencing coupled with available nematode transcriptomics and genomics data allows identification of novel effectors, thereby expanding the current effector cyst nematode repertoire (Vieira and Gleason, 2019). Also, the prediction of dorsal gland promoter elements or specific motifs such as the dorsal gland box (DOG box; Eves-van den Akker and Birch, 2016; Eves-van den Akker et al., 2016) will contribute to the identification of pioneer cyst nematode effectors. Effector libraries can be subsequently used in screening approaches on resistant plant backgrounds with known *R* genes to find the corresponding Avirulence factors. In addition, such so called effectoromics approaches can also enable the identification of novel *R* genes based on the activation of a specific hypersensitive response upon screening of natural or breeding populations. So in conclusion, expanding the repertoire of R and Avr proteins in future research is a key step to obtain novel insights on the different molecular and cellular mechanisms underlying cyst nematode resistance and virulence. Moreover, it will contribute to a better understanding on how cyst nematodes are detection by the plant’s immune system, but also how cyst nematodes have evolved mechanisms to evade host-specific defense mechanisms to increase their virulence. Ultimately, this knowledge can be exploited to develop improved control strategies to counteract cyst nematodes in crops.

## Author Contributions

QZ and AG contributed to the outline of the study. All authors contributed to manuscript writing, revision, reading, and approved the submitted version.

### Conflict of Interest

The reviewer JJ declared a past co-authorship with one of the authors AG to the handling editor.

The remaining authors declare that the research was conducted in the absence of any commercial or financial relationships that could be construed as a potential conflict of interest.
